# A seven day running training period increases basal urinary hepcidin levels as compared to cycling

**DOI:** 10.1186/1550-2783-11-14

**Published:** 2014-04-09

**Authors:** Marc Sim, Brian Dawson, Grant J Landers, Dorine W Swinkels, Harold Tjalsma, Erwin T Wiegerinck, Debbie Trinder, Peter Peeling

**Affiliations:** 1 School of Sport Science, Exercise and Health, The University of Western Australia, Crawley, Western Australia, Australia; 2 Department of Laboratory Medicine, Laboratory of Genetic, Endocrine and Metabolic Disease, Radboud University Medical Centre, Nijmegen, The Netherlands; 3 School of Medicine and Pharmacology, The University of Western Australia, Fremantle Hospital, Fremantle, Western Australia, Australia; 4 Harry Perkins Institute for Medical Research, Nedlands, Western Australia, Australia

**Keywords:** Iron deficiency, Weight-bearing exercise, Non-weight-bearing exercise, Cytokines

## Abstract

**Background:**

This investigation compared the effects of an extended period of weight-bearing (running) vs. non-weight-bearing (cycling) exercise on hepcidin production and its implications for iron status.

**Methods:**

Ten active males performed two separate exercise training blocks with either running (RTB) or cycling (CTB) as the exercise mode. Each block consisted of five training sessions (Day 1, 2, 4, 5, 6) performed over a seven day period that were matched for exercise intensity. Basal venous blood samples were obtained on Day 1 (D1), and on Recovery Days 3 (R3) and 7 (R7) to assess iron status, while basal and 3 h post-exercise urinary hepcidin levels were measured on D1, D2, D6, as well as R3 and R7 (basal levels only) for each condition.

**Results:**

Basal urinary hepcidin levels were significantly elevated (p ≤ 0.05) at D2, R3 and R7 as compared to D1 in RTB. Furthermore, 3 h post-exercise urinary hepcidin levels on D1 were also significantly higher in RTB compared to CTB (p ≤ 0.05). In CTB, urinary hepcidin levels were not statistically different on D1 as compared to R7. Iron parameters were not significantly different at D1 compared to R3 and R7 during both conditions.

**Conclusions:**

These results suggest that basal hepcidin levels may increase over the course of an extended training program, especially if a weight-bearing exercise modality is undertaken. However, despite any variations in hepcidin production, serum iron parameters in both RTB and CTB were unaffected, possibly due to the short duration of each training block. In comparing running to cycling, non-weight-bearing activity may require more training sessions, or sessions of extended duration, before any significant changes in basal hepcidin levels appear. Chronic elevations in hepcidin levels may help to explain the high incidence of iron deficiency in athletes.

## Introduction

Iron plays a number of critical roles within the body, including oxygen (O_2_) transport and energy production [1]. Specific to athletes, iron status may be compromised as a result of exercise-induced sweating, hemolysis, hematuria and gastrointestinal bleeding (see [2] for review). Recent work has suggested that post-exercise increases in the iron regulatory hormone hepcidin may also alter iron metabolism [3-9]. Hepcidin is a peptide hormone that plays a key role in regulating iron metabolism. Elevated hepcidin levels degrade the ferroportin export channels on the surface of macrophages and the intestinal duodenum, resulting in a reduction in iron recycling (by macrophages from senescent erythrocytes) and absorption from the intestine, respectively [10,11]. Presently, numerous studies have reported that hepcidin levels peak 3 h post-exercise [3-9]. These studies have attributed such a response to exercise-induced increases in the inflammatory cytokine interleukin-6 (IL-6).

To date, most studies have used running-based protocols to investigate the post-exercise hepcidin response [3-6,8,9]. Until recently, the use of alternate modalities such as cycling remained unclear. However, Troadec et al. [12] recently reported that a 45 min low intensity cycling trial (60% of heart rate reserve) did not influence post-exercise IL-6 and hepcidin levels. Subsequently, Sim et al. [7] reported that IL-6 and hepcidin levels were significantly elevated in the post-exercise period after high (interval) and low (continuous) intensity running and cycling. Of interest, serum iron was not significantly elevated immediately after low intensity cycling, suggesting that non-weight bearing exercise may have the potential to reduce the degree of exercise-induced hemolysis, similar to the findings of Telford et al. [13]. Although these studies have provided some insight into the benefits of using cycling as an alternate exercise modality, it remains unclear whether such differences may improve iron status over an extended training period.

Currently, limited studies have attempted to examine how exercise might affect post-exercise hepcidin production over an extended period, and what the implications may be for iron status. Recently, Auersperger et al. [14] reported that serum hepcidin and ferritin decreased in athletes adopting an eight week interval running program. In addition, McClung et al. [15] showed that nine weeks of basic combat training (BCT) compromised numerous iron parameters in female soldiers. On the contrary, McClung et al. [16] reported that seven days of training (military specific exercise and ski marching) elevated hepcidin levels without affecting iron status in male soldiers. Of importance, the iron status of an athlete may also dictate both the pre-exercise levels of hepcidin, and the magnitude of hepcidin response to an acute exercise stimulus (e.g. serum ferritin <30 μg.L^−1^, hepcidin suppressed) [17].

Considering that the aforementioned investigations used mainly weight-bearing activity (that may have increased the degree of exercise-induced hemolysis), it remains to be investigated how accumulated bouts of weight-bearing (running) vs. non-weight-bearing (cycling) exercise may impact iron status over time. Additionally, previous investigations [14-16] have only measured basal hepcidin levels; however, the acute post-exercise hepcidin response over consecutive exercise bouts currently remains unknown. As such, this study set out to compare the effects of a seven day period of running vs. cycling exercise on hepcidin production and iron status in active individuals.

## Methods

Ten active males participated in this study [age = 24 ± 1 y, body mass = 70.5 ± 3.2 kg, stature = 175.9 ± 2.6 cm, running peak oxygen uptake (VO_2peak_) = 58.0 ± 2.0 ml.kg^−1^.min^−1^, cycling VO_2peak_ = 49.7 ± 1.8 ml.kg^−1^.min^−1^]. At the time of recruitment, participants were performing a minimum of three exercise training sessions per week. The sample size was determined via customised computer software (GPOWER Version 2, Department of Psychology, Bonn University, Bonn, Germany) using effect sizes (ES) attained from similar research [3-7,18]. A sample size of 10 was recommended to yield a power of 0.90 at a significance level of p ≤ 0.05. When recruited, all participants had a healthy iron status (serum ferritin = 79.3 ± 15.0 μg.L^−1^, transferrin saturation = 33 ± 3%), and were not taking any iron supplements. Prior to participation, written consent was obtained with approval granted by the Human Ethics Committee of The University of Western Australia (RA/4/1/5636).

### Experimental overview

**Figure 1:**
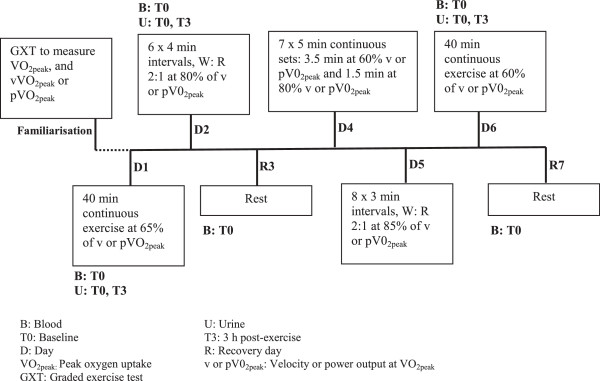
Diagrammatic representation of the running and cycling training blocks.

Participants attended the laboratory for two separate running (RTB) and cycling (CTB) exercise training blocks over a three week period. Each training block lasted for seven days, consisting of five exercise training sessions and two rest days. The training sessions were designed to engage both the aerobic and anaerobic energy systems, and consisted of a variety of training types (e.g. low intensity aerobic, fartlek, and intervals). Participants were assigned to RTB or CTB in a randomised, counter-balanced order. Subsequently, in the week prior to each training block, a familiarisation session consisting of a graded exercise test (GXT) was performed on a motorised treadmill or cycle ergometer to determine each individual’s running and cycling VO_2peak_, maximum HR (HR_max_), and the corresponding velocity (vVO_2peak_) or power output (pVO_2peak_). During each seven day period, exercise training was performed on Day One (D1), Two (D2), Four (D4), Five (D5) and Six (D6), while Days Three (R3) and Seven (R7) were recovery days (Figure 1). After completing their first training block, participants had a seven day recovery period before they started the subsequent condition. In addition, no manual labour or exercise training was performed outside of the experimental protocol, and participants were asked to keep their physical activity levels to a minimum during the seven days of recovery between conditions.

For the duration of both conditions, all exercise sessions started between 0700–0800 each day, and participants were provided with 300 ml of water to be consumed *ad-libitum.* For RTB and CTB, baseline venous blood samples were taken on three separate occasions, which included D1, R3 and R7. Finally, urine samples were obtained on arrival (baseline) and 3 h post-exercise on D1, D2 and D6, as well as R3 and R7 (baseline only). All baseline venous and urine samples were obtained between 0700 and 0800 to minimise diurnal variation.

### Experimental procedures

#### Graded exercise test

The running GXT was conducted on a motorised treadmill (VR 3000, NuryTech Inc, Germany) utilising 3 min exercise and 1 min rest periods. The initial speed was 10 km.h^−1^, with subsequent 1 km.h^−1^ increments over each exercise period until volitional exhaustion. The cycle GXT was conducted in a similar fashion (3 min exercise: 1 min rest), and performed on a calibrated wind-braked ergometer (Evolution Pty. Ltd., Melbourne, Australia), using customised data collection software (Cyclemax, School of Sport Science, Exercise & Health, The University of Western Australia). The initial workload was 100 W, with increments by 40 W every 3 min. Both HR and ratings of perceived exertion (RPE) were recorded during the final 10 s of each workload. Expired air was analysed for O_2_ and CO_2_ concentrations (Ametek Gas Analysers, Applied Electrochemistry, SOV S-3A/1 and COV CD-3A, Pittsburgh, PA), with the analysers calibrated pre-test and verified post-test using certified gravimetric gas mixtures (BOC Gases, Chatswood, Australia). Ventilation was recorded every 15 s via a turbine ventilometer (Vacumed Universal Ventilation Meter, 17125, Ventura, CA), calibrated before, and verified after exercise using a 1 L syringe in accordance with the manufacturer’s specifications. Peak oxygen consumption was determined by summing the four highest consecutive 15 s VO_2_ values.

### Exercise trials

#### Running training

All running sessions were conducted outdoors on a marked ~600 m track, consisting of grass (300 m) and bitumen road (300 m) surface. Participants were provided with a Global Positioning System (GPS) enabled watch (Garmin Forerunner 110, Garmin International Inc, Kansas, USA) to assist in pace-maintenance, and strictly adhered to the stipulated velocity for each session based on their predetermined vVO_2peak_ (see Table 1) attained during the GXT (mean vVO_2peak_: 15.0 ± 0.3 km.h^−1^, see Figure 1 for a comprehensive breakdown of each running session). These sessions were performed under comfortable environmental conditions (Dry Globe temperature: 27.0 ± 0.8°C, Relative Humidity: 58 ± 3%, Wind Speed 4.9 ± 0.8 km.h^−1^).

**Table 1:** **Mean (±SEM) heart rate (HR) expressed as a percentage of the maximum HR (%HR**_**max**_**) attained during each respective graded exercise test, ratings of perceived exertion (RPE) and the prescribed intensity for each exercise trial during the running (RTB) and cycling (CTB) training blocks**

	**Day 1**		**Day 2**		**Day 4**		**Day 5**		**Day 6**	
	**RTB**	**CTB**	**RTB**	**CTB**	**RTB**	**CTB**	**RTB**	**CTB**	**RTB**	**CTB**
**%HR**_**max**_	84	84	89	89	86	85	89	89	78	78
	(1)	(1)	(1)	(2)	(1)	(1)	(1)	(1)	(1)	(2)
**RPE**	12^a^	14	13^a^	15	12^a^	14	14^a^	16	11^a^	12
	(1)	(0)	(0)	(0)	(0)	(0)	(0)	(0)	(0)	(0)
**Prescribed exercise intensity (kph or watts)**	9.8	198	12.0	243	9.0/12.0^+^	182/243^+^	12.8	258	9.0	182
	(0.2)	(7)	(0.3)	(8)	(0.2/0.3)	(6/8)	(0.3)	(9)	(0.2)	(6)

^a^Significantly different to CTB on the corresponding day.

^+^(recovery/effort speed or power).

#### Cycling training

All cycling sessions were performed in a laboratory (Dry Bulb Temperature: 25.1 ± 0.1°C, Relative Humidity: 52 ± 0%) on a calibrated wind-braked ergometer (Evolution Pty. Ltd., Melbourne, Australia), using customised data collection software (Cyclemax, School of Sport Science, Exercise & Health, The University of Western Australia). This software program provided instantaneous and mean power feedback, which enabled participants to perform the training sessions based on their pVO_2peak_ (Table 1) attained during the GXT (mean pVO_2peak_: 304 ± 10 W, see Figure 1 for a comprehensive breakdown of each cycle session).

### Heart rate, ratings of perceived exertion

Heart rate and RPE were collected at 5 min intervals (when the exercise task was of a continuous nature; D1, D4 and D6) or at the end of each interval effort (for days where interval training was performed; D2 and D5) during the training sessions for RTB and CTB. Heart rate was measured using a Garmin HR monitor (Garmin Forerunner 110, Garmin International Inc, Kansas, USA), while RPE adopted Borg’s 6–20 scale (6 = no exertion to 20 = maximal exertion) [19].

### Food intake

Participants completed a food diary for the entire seven days of RTB and CTB. They were required to record detailed information on food type and serving size. To standardise the food intake between the different training weeks, participants were instructed to replicate their daily eating habits for the duration of the study. This data was then entered into a commercial software program (Foodworks 2007, Version 5, Service-pack 1) to obtain the percentage of macronutrient (carbohydrates, fats, protein), food iron content and total kilojoule (kj) intake.

### Blood collection and analysis

After participants lay down for a minimum of 5 min, venous blood was collected via venepuncture of an antecubital forearm vein into two 8.5 ml SST II gel vacutainers (BD, PL6 7BP, United Kingdom). Subsequently, the blood clotted for 60 min at room temperature, before being centrifuged at 10°C and 3000 rpm for 10 min. The serum supernatant was divided into 1 ml aliquots and stored at −80°C until analysis. Serum iron studies and high sensitivity C-reactive protein (CRP) were measured at Royal Perth Hospital Pathology Laboratory (Pathwest, Perth, Western Australia, Australia). Serum iron was measured using the Architect analyser (c1600210), and determined using an Iron Reagent (Sentinel Diagnostics, Milano, Italy). Coefficient of variation (CV) for iron determination at 12.01 and 43.35 μmol.L^−1^ was 1.73 and 0.61%, respectively. Serum ferritin levels were determined using an Architect analyser (1SR06055) and a Ferritin Reagent (Abbott Diagnostics, Illinois, USA). The CV for ferritin determination at 28.62, 223.05 and 497.85 μg.L^−1^ was 4.58, 4.46 and 4.36%, respectively. Transferrin was measured using Architect analyser (c1600210), and determined using a Transferrin Reagent (Abbott Diagnostics, Abbott Laboratories Abbott Park, IL 60064 USA). The CV for transferrin determination at 19.29, 32.23, 42.60 μmol.L^−1^ was 1.78 and 1.19, 1.39%, respectively. The CRP was measured using an Architect analyser (c16000), and determined using a CRP Vario Reagent (Abbott Diagnostics, Abbott Laboratories, Abbott Park, IL 60064, USA). The CV for CRP determination at 5.89 and 24.76 mg.L^−1^ was 2.08 and 2.03%, respectively.

### Urine collection and analysis

Urine samples were collected in 75 ml sterilised containers and were centrifuged at 10°C and 3000 rpm for 10 min. The supernatant was divided into 1 ml aliquots and stored at −80°C until analysis. Urinary hepcidin-25 was measured at the Department of Clinical Chemistry, Radboud University Nijmegen Medical Centre, the Netherlands, by a combination of weak cation exchange chromatography and time-of-flight mass spectrometry (WCX-TOF MS) [20,21]. An internal standard (synthetic hepcidin-24; custom made Peptide International Inc.) was used for quantification. Peptide spectra were generated on a Microflex LT matrix-assisted laser desorption/ionisation TOF MS platform (Bruker Daltonics). Values were normalised to urine creatinine values and reported in nmol/mmol creatinine. The lower limit of detection was 0.1 nmol.L^−1^ with an intra-run and inter-run CV of 3 to 7% and 10 to 13%, respectively [22].

### Statistical analysis

Results are expressed as mean and standard error of the mean ± SEM. Repeated measures ANOVA analysed time, trial and time*trial effects of the different RTB and CTB sessions on various serum iron and inflammatory parameters, as well as urinary hepcidin levels. Post-hoc paired samples t-tests were used to determine where specific trial differences existed, using an alpha level set at p ≤ 0.05. Cohens-d ES were also calculated (<0.4 = *small*, 0.4-0.8 = *moderate*, >0.8 = *large*).

## Results

### Heart rate and ratings of perceived exertion

Mean HR for each trial was expressed as a percentage of the maximum HR (HR_max_) attained during the running and cycling GXT; which were 193 ± 3 and 186 ± 3 bpm, respectively. Mean percentage of HR_max_ was not significantly different between any of the running and cycling training sessions on their corresponding days (Table 1). Mean RPE was significantly higher (p ≤ 0.05) in all cycle training sessions as compared to running on their corresponding days (Table 1).

### Food intake

Daily kJ for RTB and CTB was 10,171 ± 305 and 10,027 ± 268 kJ, respectively. For RTB, the percentage composition of daily kJ intake for carbohydrates, fats and proteins was 47 ± 2, 27 ± 2 and 22 ± 1%, respectively. For CTB, the percentage composition of daily kJ intake for carbohydrates, fats and proteins was 49 ± 2, 25 ± 2 and 22 ± 1%, respectively. The daily food iron content for RTB and CTB was 6.7 ± 0.5 and 6.7 ± 0.6 mg, respectively. Daily energy intake, the percentage composition of carbohydrates, fats and proteins, as well as food iron content were not different between conditions (p > 0.05).

### Blood parameters

Blood parameters are displayed in Table 2. No time or trial effects were recorded for serum ferritin and iron, as well as transferrin saturation on D1, R3 and R7 for both RTB and CTB. Although no trial effects existed for CRP, time effects revealed that CRP levels were significantly lower (p ≤ 0.05) at R7 as compared to D1 during CTB.

**Table 2:** Mean (±SEM) baseline serum ferritin, iron, transferrin saturation and C-reactive protein (CRP) at Day 1, Recovery Days 3 and 7 in the running (RTB) and cycling (CTB) training blocks

**Blood Parameters**	**RTB**			**CTB**		
	**Day 1**	**Recovery 3**	**Recovery 7**	**Day 1**	**Recovery 3**	**Recovery 7**
**Serum Ferritin (μg.L**^**−1**^**)**	79.3	82.6	84.2	84.7	82.4	77.9
	(15.0)	(16.0)	(13.7)	(17.4)	(15.5)	(15.5)
**Serum Iron (μmol.L**^**−1**^**)**	19.6	20.3	17.5	15.8	22.6	17.5
	(2.0)	(1.5)	(2.0)	(1.0)	(2.8)	(1.6)
**Transferrin Saturation (%)**	33	34	30	26	37	29
	(5)	(2)	(4)	(2)	(4)	(2)
**CRP (mg.L**^**−1**^**)**	1.08	1.10	0.91	1.17	1.12	0.75^a^
	(0.35)	(0.34)	(0.33)	(0.38)	(0.38)	(0.28)

^a^Significantly different to CTB Day1.

### Urinary hepcidin

Urinary hepcidin levels on the exercise days (D1, D2, D6) are displayed in Table 3. On D1, significant time and interaction effects (p ≤ 0.05) were demonstrated, with post-hoc analysis revealing that hepcidin levels were significantly higher 3 h post-exercise as compared to baseline during RTB (p ≤ 0.05), which was supported by a large ES (d = 1.68). Furthermore, 3 h post-exercise hepcidin levels were significantly higher (p ≤ 0.05) during RTB as compared to CTB (d = 0.68, *moderate*). For D2, there were no significant main effects, although a large ES (d = 0.99) suggested that hepcidin levels may be increased 3 h post-exercise when compared to baseline for RTB. Additionally, baseline hepcidin levels were significantly higher at D2 as compared to D1 for RTB (p ≤ 0.05). For D6, no significant main effects were again recorded. However, large ES suggested hepcidin levels may increase 3 h post-exercise as compared to baseline in both RTB (d = 1.69) and CTB (d = 0.99). Basal urinary hepcidin levels for D1, R3 and R7 are displayed in Table 4. No trial effects were recorded between days, but time effects revealed that hepcidin levels were significantly higher at R3 (p = 0.010; d = 0.79, *moderate*) and R7 (p = 0.016; d = 0.49, *moderate*) as compared to baseline in RTB. Additionally, a large ES (d = 1.26) suggested that basal hepcidin levels were higher at R7 than D1 during CTB.

**Table 3:** Mean (±SEM) for urinary hepcidin levels at baseline (T0) and 3 h post-exercise (T3) during the exercise days for the running (RTB) and cycling (CTB) training blocks

**Urinary hepcidin (nM.mmol Cr**^**−1**^**)**				**p-values**			**Effect sizes**		
		**T0**	**T3**	**Trial**	**Time**	**Interaction**	**T0-T3**	**T0: RTB-CTB**	**T3: RTB-CTB**
**Day 1**	**RTB**	0.46	1.19^a^	0.179	0.002	0.014	1.68	0.15	0.68
		(0.14)	(0.26)						
	**CTB**	0.52	0.64^b^				0.63		
		(0.06)	(0.10)						
**Day 2**	**RTB**	0.76^c^	1.38	0.524	0.245	0.190	0.99	0.14	0.54
		(0.20)	(0.37)						
	**CTB**	0.85	0.84				0.02		
		(0.24)	(0.28)						
**Day 6**	**RTB**	0.71	0.93	0.173	0.171	0.505	1.69	0.29	0.25
		(0.04)	(0.16)						
	**CTB**	0.43	0.80				0.99		
		(0.12)	(0.28)						

^a^Significantly different to T0.

^b^Significantly different to RTB Day 1, T3.

^c^Significantly different to RTB Day 1, T0.

**Table 4:** Mean (±SEM) urinary hepcidin levels at baseline (T0) on Day 1 and Recovery days 3 and 7 for the running (RTB) and cycling (CTB) training blocks

**Urinary hepcidin (nM.mmol Cr**^**−1**^**)**			**p-values**			**Effect sizes**		
		**T0**	**Trial**	**Time**	**Interaction**	**RTB -CTB**	**Day 1-Recovery 3, 7**	**Recovery 3-7**
**Day 1**	**RTB**	0.62	1.000	0.047	0.365	0.15	-	-
		(0.20)						
	**CTB**	0.56						
		(0.10)						
**Recovery 3**	**RTB**	0.80^a^				0.28	0.79	-
		(0.17)						
	**CTB**	0.64					0.64	
		(0.18)						
**Recovery 7**	**RTB**	0.67^a^				0.20	0.49	0.24
		(0.14)						
	**CTB**	0.76					1.26	0.21
		(0.18)						

^a^Significantly different to RTB Day1.

## Discussion

The results of this investigation suggest that acute bouts of running (as compared to cycling) performed over a seven day period have the ability to significantly increase basal urinary hepcidin levels. Hepcidin levels were also significantly elevated 3 h post-exercise compared to baseline on D1 of RTB, with strong ES evident to suggest acute increases in hepcidin levels in the post-exercise recovery period after the majority of all training sessions. Such findings concur with the results of previous research [3-9]. Despite this, it should also be considered that any changes in basal hepcidin levels at R7 as compared to D1 did not appear to directly impact any iron parameters in either condition.

### Hepcidin and inflammation

Previously, it has been suggested that elevated hepcidin levels in the post-exercise recovery period may alter iron metabolism in athletes [3-9]. These studies have highlighted the role of the inflammatory cytokine IL-6 and hemolysis in this process, suggesting that chronically elevated hepcidin levels may explain the high incidence of iron deficiency commonly reported in athletes. Such a proposition appears plausible based on the results of the current investigation, since basal hepcidin levels were significantly higher during RTB at D2, R3 and R7, compared to D1. Furthermore, although not statistically significant, moderate to large ES suggest basal hepcidin levels appeared higher at R3 (d = 0.64) and R7 (d = 1.26) compared to baseline in CTB.

Despite the large ES for hepcidin to increase, the inflammatory marker CRP was not significantly higher at R3 and R7 as compared to D1 in both conditions, suggesting no accumulated increases in inflammation. Typically, exercise-induced hepcidin production has been linked specifically to elevations in IL-6, which peaks immediately post-exercise [3-9,18]. Although IL-6 was not measured here, CRP synthesis can be stimulated by increases in pro-inflammatory cytokines such as IL-6, IL-1 and tumor necrosis factor (TNF)-alpha [23,24], and as such, CRP was selected as a surrogate measure of inflammation. Despite CRP levels being previously reported to be elevated up to 24 h post-exercise [6], this was not observed in the current investigation. However, in agreement with these results, previous investigations have shown IL-6 and CRP to be lower after nine weeks of BCT in female soldiers [25]. Such an outcome suggests that any exercise-related inflammatory processes that were evident here were quickly returned to baseline levels during the subsequent recovery period.

Recently, Auersperger and colleagues [14] investigated the effects of an eight week continuous or interval running program on hepcidin, inflammatory markers and iron status in females. These authors reported that serum hepcidin levels in both groups were significantly lower (compared to baseline) after the first three week period, as well as one week after completing a competitive race at the end of the study (10 or 21 km). Additionally, Ma et al. [26] reported that basal serum hepcidin and IL-6 gene expression were not significantly different between female distance runners and matched controls. The contradictory results of Auersperger et al. [14] and Ma et al. [26] to those of the current investigation may have been influenced by two factors: (a) their populations declining (or pre-existing poor) iron status during the training period, and (b) hormonal fluctuations in the menstrual cycle. Previously, these factors have both been raised as potential mechanisms that could attenuate hepcidin production (for review see [27]). For example, serum ferritin has recently been reported to dictate hepcidin activity in athletes [17]. Here, Peeling and colleagues [17] demonstrated that low serum ferritin levels (<30 μg.L^−1^) were linked to the suppression of pre-exercise levels of hepcidin, and the magnitude of hepcidin response to an acute exercise stimulus. Additionally, concerns were raised for individuals with ‘suboptimal’ iron stores (serum ferritin 30–50 μg.L^−1^), as the post-exercise hepcidin response in these individuals was still evident after 3 h of recovery, at a similar magnitude to those athletes presenting with more healthy iron stores. Considering that both the running and control groups in Ma et al. [26] presented with poor iron stores (at the time of biological sampling; serum ferritin of < 35 ug.L^−1^), they would also be classified as Stage One Iron Deficient according to numerous published guidelines for athletes [2,28]. Consequently, these previous findings may only be relevant to populations displaying a poor iron status.

Karl et al. [25] also reported that serum hepcidin levels were unchanged in female soldiers who had performed a nine week BCT training program while receiving an iron fortified food bar (twice daily) or a placebo equivalent. However, when soldiers were regrouped according to their iron status (either Normal [NORM], Iron Deficient [ID] or Iron Deficient Anemic [IDA]), post-BCT basal hepcidin levels were significantly lower in IDA as compared to NORM, while the ID group showed similar decreases without reaching significance (p = 0.06). Most importantly, it should be highlighted that during the aforementioned investigations, basal hepcidin samples were obtained at the end of specific training phases [14,25] or at a single time point [26], without measuring any acute changes over the course of the training period. In our investigation, basal hepcidin levels were measured on five occasions (D1, D2, R3, D6, R7), in addition to 3 h post-exercise samples (D1, D2, D6) to highlight the acute hepcidin response. Additionally, this is the first investigation to explore if any benefits associated with iron metabolism might be present after completing a series of non-weight-bearing exercise (cycling) sessions as compared to weight-bearing activity (running) in active males.

Numerous exercise investigations have explored the hepcidin response acutely [3-9], showing that the hormone levels are significantly elevated 3 h post-exercise (as compared to baseline) after each exercise session. However, such a response was only recorded here on D1 of RTB, with moderate to large ES recorded for the majority of the other running and cycling sessions. Such findings may be linked to the accumulation of acute post-exercise increases in hepcidin (supported by moderate to large ES), which may be responsible for raising basal levels (as seen at R3 and R7). As such, elevated basal hepcidin activity may have reduced the magnitude by which hepcidin increases acutely, as a result of the exercise task. Despite this, it would appear that acute bouts of running (and to a lesser degree cycling) performed over a seven day period, may still have the ability to increase basal urinary hepcidin levels (e.g. D1 vs. R7).

In consideration of this finding, the accumulation of hepcidin levels over an extended training program might help to explain the high incidence of iron deficiency commonly observed amongst athletes. Such a proposition is supported by McClung et al. [16], where four days of military specific training followed by a three day cross-country ski march performed by male soldiers (~20 km/day, with 45 kg backpacks), caused an increase in serum IL-6 and hepcidin. This increase in hepcidin activity after their military training would be comparable to the significant hepcidin increases recorded at R7 (as compared to D1 in RTB). However, since training volume has been shown to influence hepcidin production [3], the findings of McClung and colleagues [16] are likely to be exacerbated in comparison to those presented here, possibly as a result of the greater training load undertaken. Furthermore, since the aforementioned investigations have only adopted weight-bearing activity [14,16,25], it is also possible that these results may be different under the influence of non-weight-bearing exercise. With this in mind, it is evident that basal hepcidin levels were likely higher at R7 as compared to D1 in the CTB. Therefore, it is possible that cycling training also has the potential to elevate basal hepcidin levels. However, given the weight supported nature of the exercise task, it might be that exercise of an extended duration, and/or additional training sessions are required before a similar magnitude of response is recorded comparative to running-based training.

Finally, although the findings of this investigation are novel and important, a limitation of this study may be perceived from the measurement of hepcidin in the urine instead of serum. Previously, it has been demonstrated that urinary hepcidin measures were substantially lower than circulating serum levels [29]. As such, serum measurements are preferable to detect small changes in hepcidin levels. However, due to the nature of the current experimental design, involving numerous sampling time points and logistical requirements for each seven day period, urinary measurements were selected as it represented the most practical option for sample collection. Regardless, it is possible that if serum hepcidin measurements were performed here instead of urine (similar to [16]), the tendency for hepcidin levels to be higher at the end of RTB and CTB may have become stronger and more consistent.

### Iron parameters

This investigation demonstrated that iron parameters (serum ferritin and iron, transferrin saturation) remained relatively stable over the course of RTB and CTB, possibly due to the relatively short duration of the experimental period (seven days), as compared to other investigations that obtained conflicting results [14,15,25].

Currently, numerous studies have highlighted the importance of maintaining optimal iron stores throughout a training program. However, a reduction in iron status over the course of an extended training period has been commonly reported [15,25,30]. McClung et al. [15] previously examined how iron parameters may be altered by BCT. These authors reported that markers of both iron storage (serum ferritin) and transport (transferrin saturation) had decreased post-BCT. In support of these findings, Di Santolo et al. [31] also suggested that athletes who performed ~11 h per week of training had reduced ferritin and transferrin saturation levels compared to sedentary controls. The discrepancy between our results and these investigations is potentially due to the shorter duration of the intervention employed here (five sessions over seven days) as compared to the substantially greater number of accumulated sessions over the two month period in other studies [15,25]. Considering that both hepcidin and iron parameters during CTB were not significantly different at R7 as compared to D1, perhaps the use of cycling (as opposed to running) may be better suited to iron deficient individuals, who are required to maintain fitness levels, while consuming iron supplements to replenish iron stores. Specifically, as hemolysis contributes towards iron loss [32], the use of non-weight bearing activity (such as cycling) to reduce hemolysis [13] may be beneficial.

Previously, Telford and colleagues [13], demonstrated significantly higher levels of hemolysis after completing an intensity matched running, as compared to cycling trial (1 h run or cycle at 75% VO_2peak_). These results were attributed to the impact forces associated with footstrike that increased hemolysis, possibly having implications for exercise-induced iron loss in athletes [32]. Similar results were also reported by Sim et al. [7], where 10 well trained male triathletes performed four separate experimental sessions consisting of high (8 × 3 min intervals at 85% v or pVO_2peak,_ W:R 2:1) and low (40 min continuous exercise at 65% v or pVO_2peak_) intensity running and cycling, with significant increases in hemolysis immediately post-exercise reported in all trials except for low intensity cycling. However, since the current investigation adopted both high and low intensity sessions during CTB (within a relatively short duration of seven days), any benefits associated with reduced hemolysis during this training period may not have been reflected by the serum iron parameters. To this end, it remains unknown if these findings may be altered over the course of an extended cycling program (e.g. >2 months).

## Conclusion

In summary, these results suggest that basal urinary hepcidin levels may be significantly elevated after a series of acute running sessions (five sessions over seven days). However, such events may not be observed if an identical cycling program is adopted*.* Perhaps, more exercise sessions, or sessions of greater duration may be undertaken with cycling as an exercise medium, before a significant increase in basal hepcidin levels is recorded. Additionally, despite any variations in hepcidin, this did not appear to influence serum iron parameters in RTB and CTB. This study supports the idea that basal hepcidin levels may increase (due to an accumulation of acute exercise-induced responses) over the course of an extended training program; although it remains to be established if such a response may compromise an individual’s ability to absorb and recycle iron, which may explain the high incidence of iron deficiency commonly reported in athletes.

## Competing interests

The authors wish to declare that no competing interests existed as part of the preparation of this manuscript.

## Authors’ contributions

MS: Study concept and design, data collection and analysis, measurement of biological samples, manuscript preparation. BD: Study concept and design, data analysis and interpretation, manuscript preparation. GL: Study concept and design, data analysis and interpretation, manuscript preparation. DS: Study concept and design, measurement of hepcidin samples, manuscript preparation. HT: Study concept and design, measurement of hepcidin samples, manuscript preparation. EW: Study concept and design, measurement of hepcidin samples, manuscript preparation. DT: Study concept and design, data analysis and interpretation, manuscript preparation. PP: Study concept and design, measurement of biological samples, data analysis and interpretation, manuscript preparation. All authors read and approved the final manuscript.
